# Knowledge apartheid in disaster risk management discourse: Is marrying indigenous and scientific knowledge the missing link?

**DOI:** 10.4102/jamba.v7i1.150

**Published:** 2015-05-07

**Authors:** Mukundi Mutasa

**Affiliations:** 1 Southern African Development Community (SADC) Secretariat, Botswana

## Abstract

Indigenous knowledge (IK) is a key component of disaster risk management (DRM) and development planning, yet it is often overlooked, with practitioners preferring to use scientific knowledge. Critics of IK have termed it archaic, primitive, a constraint to development and inferior to scientific knowledge, which has contributed to its widespread marginalisation. However, smallholder farmers in rural Zimbabwe have utilised IK for generations, especially in predicting rainfall patterns and managing drought conditions, showing that IK can be a useful tool in DRM. This article presents findings from research on drought vulnerability and coping conducted in Zimbabwe's Buhera and Chikomba districts in 2009, particularly relating to utilisation of IK in smallholder farming communities, and argues that unless IK is documented and preserved, its marginalisation will persist. The research followed a mixed-methods approach whereby both quantitative and qualitative data were collected and analysed. Whilst smallholder respondents were randomly selected for household surveys, snowball sampling was employed for key informant interviews. Respondents indicated that they utilised some indigenous rainfall pattern predictions gained from observing and interpreting plant and animal behaviour. Some cultural practices that were critical to development and utilisation of certain IK were also threatened with extinction. The article argues for 'marrying’ IK and scientific knowledge, in the hope that the two will offset each other's weaknesses, resulting in some kind of hybrid knowledge that will be critical for promoting sustainable agricultural production in Zimbabwe. However, this is not for disregard the challenges associated with knowledge hybridisation, as these two types of knowledge are grounded on differing foundations.

## Introduction

The growing contagion of critics questioning the integrity of indigenous knowledge (IK) coincides with an equally widespread crusade supporting its integration in disaster risk management (DRM) and development planning. In fact, ‘the use of indigenous knowledge in development has become a kind of mantra’ (Briggs 2005:99). Agrawal (1995:413) concurs, arguing that IK has become ‘[*one*] of the more glamorous phrases that has now begun to colonize the lexicon of development practitioners and theorists alike.’ IK is accumulated over years of interaction with the natural environment (Hewitt 2012; United Nations Environment Programme [UNEP] 2008).

In the Zimbabwean context, smallholder farmers have relied on such knowledge for centuries, applying it mainly for survival in the wake of increased frequency and severity of droughts and other hazards. However, full utilisation of such knowledge is threatened by its marginalisation compared to modern or scientific knowledge. This is mainly because IK is sometimes viewed as archaic, backward, inefficient, primitive and uncivilised (Agrawal 1995; Briggs 2005; Kelkar 2007; Nygren 1999). Meanwhile, the tendency by the research community to eulogise scientific rainfall predictions at the expense of indigenous ways of predicting and understanding rainfall patterns can be argued to be further contributing to IK's peripherisation. Some scholars have even suggested that IK is at ‘risk of becoming extinct’ (Ngulube, Masuku & Sigauke 2011:266).

This article contributes to the growing discourse of IK systems; explores the usage of such knowledge by smallholder farmers in rural Zimbabwe; analyses the factors that contribute to marginalisation of IK; identifies the challenges that proponents of IK face in advocating its utilisation; and attempts to provide recommendations to counteract IK marginalisation in Zimbabwe.

### Defining indigenous knowledge

Knowledge involves experience and skills that people gain from interacting with information, and can be explicit, tacit or implicit (Powell 2003). IK is developed by a group of local people and passed on from generation to generation, essentially becoming part of a generation's inheritance. It is indigenous to a selected geographical location and its inhabitants. This knowledge is often not documented, yet it is an important resource facilitating community survival and development. However, for survival to be fully achieved, such knowledge needs to be combined with people's access and use rights to other resources, such as assets and income.

Nyong, Adesina and Osman Elasha (2007:792) define IK as ‘institutionalized local knowledge that has been built upon and passed on from one generation to the other by word of mouth’. According to the UNEP (2008), IK is generated over a period of time through a people's close interaction with nature. There are distinct characteristics associated with this type of knowledge, namely it is unique to a particular locality, is generated by a local people, is passed on from generation to generation, relates to the interaction between that group of people and nature in their locality, and is largely undocumented.

A plethora of terms associated with IK has been widely used in literature. These include local knowledge, traditional knowledge, indigenous traditional knowledge, indigenous technical knowledge, peasants’ knowledge, traditional environmental knowledge, folk knowledge, people's science, ethnoscience, local science, traditional science, village science, and rural knowledge (Mercer 2012:98; UNEP 2008:21; Williams & Muchena 1991:52). In this article, the term IK is preferred unless the literature quoted specifically refers to the different terminologies listed above.

A wide range of literature has shown the distinct differences between IK and scientific knowledge. According to Nygren (1999:267), IK ‘has been represented as something in opposition to modern knowledge.’ This has contributed to the marginalisation of IK, as it is seen as opposing logic and has been viewed as ‘part of a residual, traditional and backward way of life’ (Briggs 2005:102). This is regardless of the value that smallholder farming communities have for years accorded to IK, particularly in Africa. For example, Nyong *et al.* (2007) argue that IK is very important and has facilitated the survival of local populations in Africa's Sahel region in the wake of climate change and variability.

### Research objectives

This article is drawn from research conducted in 2009 whose key objectives were to understand community vulnerability to droughts in the Buhera and Chikomba districts of Zimbabwe, as well as to explore various coping mechanisms employed by the ‘at risk’ populations to cope with adversity. IK came out prominently as a tool to predict rainfall patterns in agricultural planning, as will be shown here.

## Research methods

The research from which this article is drawn utilised both qualitative and quantitative methods, an approach termed mixed-methods research. This approach ‘attempts to combine the advantages of quantitative and qualitative methods and to avoid their disadvantages’ (Bless, Higson-Smith & Sithole 2013:58) and aims to offset ‘the weaknesses of one [*research*] method by applying an alternatively better method’ within the same research (Mutasa 2010:29). Quantitative data collection was largely embedded within qualitative data collection, playing more of a supportive role to qualitative data in a process termed embedded mixed-methods research (Plano-Clark *et al.* 2008).

The research utilised semi-structured questionnaires and key informant interviews for the primary research, while secondary literature was consulted for information on historical experiences with droughts in Zimbabwe and the application of different knowledge types, especially by farming communities.

The research was conducted in the Buhera and Chikomba districts of Zimbabwe, targeting mainly smallholder farmers involved in dryland agriculture. The respondents were randomly selected, offering everyone ‘an equal opportunity of inclusion in the sample’ (Bryman 2008:171). Thirty six semi-structured questionnaires were administered in Buhera and 39 households were surveyed in Chikomba. In addition, the key informants interviewed in the communities totalled 11 for Buhera and 12 for Chikomba. Snowball sampling was employed for interviews with informants in humanitarian organisations and government departments, and theoretical saturation (whereby no new or relevant data emanates from new interviews) was employed to gauge when the collected data on the subject under study were exhaustive.

### Scope of the study

Buhera and Chikomba districts, falling in Manicaland and Mashonaland East provinces respectively, are among the more than 60 administrative districts in Zimbabwe. Agriculture in the two bordering districts is mainly smallholder and rain-fed. Rains are highly unpredictable, exposing the agricultural sector and people's livelihoods to the vicissitude of unreliable weather patterns.

**FIGURE 1:**
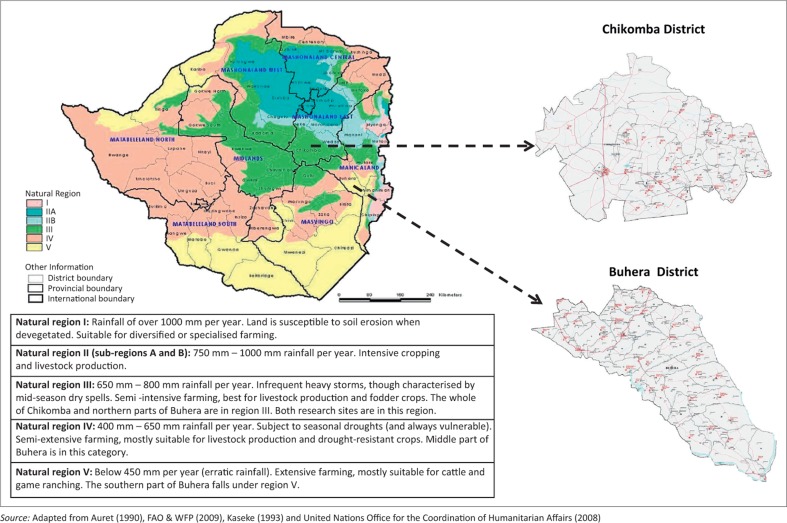
Zimbabwe's agro-ecological regions and their rainfall and agricultural characteristics. *Source:* Adapted from Auret (1990), FAO & WFP (2009), Kaseke (1993) and United Nations Office for the Coordination of Humanitarian Affairs (2008)

Zimbabwe is divided into five agro-ecological regions (Figure 1). Chikomba and northern parts of Buhera lie in semi-intensive agro-ecological region III, which experiences mid-season dry spells. However, Buhera is unique in that the characteristics of the bottom three agro-ecological regions (III, IV and V) are experienced across the whole district. The most southern parts of Buhera are often regarded as perennially food insecure, with the whole district identified as one of the poorest in Zimbabwe. In fact, the further south in the district one goes, the more food insecure the communities generally become (Gundry *et al.* 1999). However, there is growing consensus within the research community in Zimbabwe that there is a need to revisit the agro-ecological regions, due to shifting climatic conditions (Chikodzi *et al.* 2013). These agro-ecological regions were developed during the colonial era, and have barely been revised since.

## Findings

The following sections present the findings from the research, focusing mainly on utilisation of IK in the communities.

### Indigenous rainfall predictions

The research unearthed some undocumented knowledge that communities in the two districts employed to predict and understand rainfall patterns. Such predictions influenced communities’ farming activities for the respective cropping season (Table 1).

**TABLE 1:** Indigenous rainfall predictions used in Buhera and Chikomba.

Predicting a wet period or wet season	Predicting a dry season or drought
Warm winter	A very cold winter
Westerly and Northerly winds	Easterly winds
High density of spider webs in a given area	Low density of spider webs
Low abundance of wild fruits	High abundance of wild fruits prior to the farming season
Bigger circular halo around the moon, known in the Shona language as *dziva*, symbolises a wet immediate period	Smaller *dziva*, or its absence, shows how dry the immediate period will be

Some respondents conceded that they were left with no choice but to rely on indigenous predictions, because they did not understand scientific weather reports from the Meteorological Services Department whenever they received them (which was very rarely). One of the reasons for lack of understanding of the scientific weather reports was that they were too technical and rarely interpreted for the majority semi-literate rural populace.

In both districts, availability of wild fruits was used as an indicator of the season's potential outcome. A low occurrence of wild fruits signalled a potential bumper harvest in the fields. According to respondents in the two districts, this was a clear indication of how the supreme deity, God, balances the availability of wild fruits and yields in the fields, with wild fruits meant to compensate for poor agricultural yields. Examples of wild fruit trees referred to during the research included *muchakata* (*Parinari curatellifolia*), *muzhanje* (*Uapaca kirkiana* or wild loquat), and *mutukutu*/*munjekenje*/*musekesa* (*Piliostigma* *thonningii*). A *muchakata* tree is one of the sacred trees in Zimbabwe and is also used for traditional rituals such as rain ceremonies.

### Culture and the production of indigenous knowledge

There are a number of cultural practices that communities in Buhera and Chikomba have engaged in for generations, such as rain ceremonies known locally as *mikwerera* (plural for *mukwerera*). According to the respondents, it is mandatory that *mikwerera* are always conducted under a sacred *muchakata* tree that has a *rushanga* or *rumhanya* (twig fence) built around it. Community leaders are key role-players in organising and conducting rain ceremonies. In addition, the traditional beer consumed during rain ceremonies is brewed only by elderly women past menopause, using traditional small grains such as finger millet and sorghum. According to respondents, tradition considers these women sexually inactive and ritually pure. It is usually during these ceremonies that spirit mediums (*masvikiro*) deliver messages from the ancestors (*midzimu*) *vis-à-vis* the type of crops to grow that particular agricultural season. Spirit mediums are believed to possess the ability to converse with the ancestors, who help them to ‘see into the future’ and communicate the message from their ancestors to the whole community. As such, *mikwerera* were seen as key to the generation of valuable IK which is passed on from generation to generation.

Respondents in the two districts indicated that although such traditional practices were continuing, they were no longer as frequently organised as in the past, and attendance has significantly dropped. One informant blamed the effects of colonisation, for example diffusion of traditional cultures by Western ones as younger generations started shunning traditional practices, and modernisation, which has plucked the young and economically active from the rural areas to urban areas as they look to earn a ‘better living’. Respondents also indicated that modernisation and religion, particularly Christianity, have made some to even question the spirit mediums’ relevance and reliability in predicting rainfall patterns. The interviewed traditional leaders claimed that this lack of belief has contributed to the ancestors turning their backs on the communities and the spirit mediums not delivering messages from the ancestors as had been the tradition. By turning their backs on the communities, it is believed that the ancestors and God are now withholding rains as punishment, resulting in an increased frequency of droughts in the districts under study.

### Every raindrop counts

Some of the respondents indicated that their experiences with an increased frequency of droughts taught them to make use of every raindrop, regardless of when it fell. The communities used to identify rain by the time it fell, and what such rain could be used for. The common rain names include:

- *mavhurachando*, which heralds the beginning of winter
- *gukurahundi*, which washes away the chaff from grain processing
- *bumharutsva*, which extinguishes raging veld fires
- *mvumiramutondo*, which facilitates the blooming of trees
- *munhuruka,* signalling the starting of the rainy and cropping season. Usually the smallholder farmers would only start cropping after *munhuruka*.

According to some respondents, such knowledge of the different types of rain was fast disappearing.

## Discussion

In the preceding section, selected indigenous rainfall predictions and the various cultural practices observed in Chikomba and Buhera were presented. This section discusses some of these traditional practices and how they have helped in producing IK and sustaining people's livelihoods in the two districts, especially in response to unpredictable rainfall patterns affecting their main source of livelihood – agriculture. Challenges associated with promoting such knowledge and proposals to better document, validate and communicate it will also be discussed.

### A case for indigenous rainfall predictions

IK is key to development planning and DRM. Communities in Buhera and Chikomba employed a plethora of techniques to predict drought seasons. The research revealed how farmers observed plant and animal behaviour, specifically spider density and wild fruit availability, as well as wind direction to predict rainfall patterns, similar to those identified by the Food and Agriculture Organization of the United Nations (FAO 2004) and Mugabe *et al.* (2010) (Table 2). Mapeta (2000:94) also claimed that ‘if they [*wild trees*] bear plenty of fruits, we know it will be a dry year’. According to the Buhera and Chikomba communities, this was evidence of how God balances wild fruits availability and crop production, thereby ensuring that there is always a constant supply of food even when agricultural production fails. This also cements the notion that religion plays a pivotal role in understanding hazards and in informing DRM activities (Chester, Duncan & Sangster 2012; Scoones *et al.* 1996).

**TABLE 2:** Selected indigenous rainfall predictions commonly employed in Zimbabwe.

Indicators predicting a good season	Indicators predicting a poor season	Indicators of when it will rain
Heavy production of tree leaves	High abundance of (wild) fruit	An early onset of rains is
Flower production on the top branches of a *mukonde (candelabra)* tree	Heavy infestation of caterpillars during springtime	Measured by how early spiders close their nests
A stork flying at very high altitude presence of a lot of birds	Late bearing and lack of *mukute/muhute (syzygium cordatum)* figs in July – September, late maturing of acacia trees along valleys	A bird singing whilst facing downwards from the top of a tree indicates that it is about to rain
Westerly winds	Heavy populations of crickets on the ground	-
Northerly winds	Strong Easterly winds between July and early November	-
Heat waves	Extended winter period	-
North-easterly winds	White frogs appear in trees	-
Prevalence of whirlwinds	Lots of thunderstorms without rains	-
Frogs turning brownish	Early rains starting from early October	-
Rain birds making a lot of noise	-	-
Butterflies seen hovering in the air from north to south starting in October	-	-

*Source*: Adapted from FAO (2004) and Mugabe *et al.* (2010)

There appeared to be a reluctant reliance on IK in some communities in the two districts, mainly because scientific rainfall forecasts were not provided on time. A lack of understanding of the provided scientific forecasts further hampered the communities’ agricultural production efforts. Faced with such challenges, communities resorted to their traditional ways of predicting rainfall patterns, although some admitted that there was no guarantee that the season would definitely turn out the way they predicted. This was probably evidence of the diminishing reliability of IK or even its misinterpretation and/or distortion due to it being passed on from generation to generation through word of mouth and being location-specific.

Given the opportunity for ready access to scientific knowledge, one would argue that it is highly likely that the people would choose scientific forecasts over indigenous rainfall predictions, as scientific forecasts are ‘tried and tested’. However, this is not to totally discredit IK, as communities have used it for centuries, which shows how rewarding it is to warrant communities’ continued reliance on it. A study in the Sahel region revealed that IK has indeed been useful in finding solutions to challenges associated with climate change and variability (Nyong *et al.* 2007).

In a situation analysis conducted by the FAO (2004) in the Limpopo River Basin it was found that whilst a lowly 3% of Zimbabwe's smallholder farmers used climate information from the Meteorological Services Department, the similarity between the indigenous systems and the contemporary seasonal predictions were striking. This identification of similarities between IK and scientific knowledge is vital for knowledge hybridisation. However, if IK is looked at entirely from the realm of social sciences and scientific knowledge from natural sciences, it will be difficult for natural scientists to find any value in IK, whilst social scientists will continue to be emotional about its perpetual denigration.

### Importance of indigenous knowledge

The importance of IK, particularly in the context of DRM, has been extensively written on. There has been a general thinking that IK is of particular importance to communities in the developing world only. However, it should be noted that even in developed countries, IK has been a key component of socio-ecological resilience, particularly to hazards such as cyclones, floods, volcanoes and earthquakes. In New Zealand, for example, the Mãori people, indigenous to Aotearoa, are argued to possess ‘unique indigenous knowledge relating to natural hazards … developed through their interaction and adaptation with the natural environment over many centuries’ (Faulkner & Becker, quoted in Hewitt 2012:89).

Although IK faces widespread criticism because of debatable misgivings, as will be discussed in subsequent sections, its importance in Zimbabwean smallholder farming communities remains unquestionable. Williams and Muchena (1991:53) opine that such knowledge ‘is unique in that it is generated in response to the natural and human conditions of a particular environment and context.’ As such, communities in the two districts under study have managed to generate the knowledge that is unique to their weather conditions, which has helped them in the absence of scientific rainfall predictions. This has resulted in a revision of cropping patterns and employment of idioms and proverbs such as ‘*ukatsvaira dura unopinza nzara* *mumba*’, meaning that one should never exhaust the grain stocks before the next harvest. Through that knowledge, households would also revise their diets in order to ensure that they would not run out of grain as they awaited the harvest season.

Survival is the main objective in times of crisis, and IK is a key contributing factor. Briggs (2005) argues that with specific reference to poor communities, IK has an edge over Western scientific knowledge. This is mainly because knowledge possessed and/or generated by the local communities ‘is tested in the context of survival, … [*is*] more or less effective in providing the means of survival, a conclusion more meaningful in the context of everyday existence’ (Briggs 2005:103). Mercer (2012) concurs, positing that it is not only scientific knowledge that has saved lives, but local knowledge has also played its part in the context of survival. This was evidenced in Buhera and Chikomba where, even in the wake of limited access to scientific rainfall predictions, the communities appeared to be ‘surviving’ through their utilisation of IK.

The debate on IK has also touched on how the knowledge can be useful in the context of sustainable development, rather than merely focusing on survival in times of adversity (Agrawal 1995; Briggs 2005). Agrawal (1995:417) opines that IK ‘is of crucial significance if one wishes to introduce a cost-effective, participatory and sustainable development process’ as it is not expensively assembled or acquired and utilises mainly local resources.

Meanwhile, the romanticisation of IK has resulted in some scholars arguing that it ‘may even have reached as status of “a new populist rhetoric”’ (Briggs 2005:100). However, one might wonder if IK is merely intended to appeal to the populous rural people, and why it should be considered rhetoric, i.e. empty yet persuasive, which Argawal (1995) also referred to. It can be argued that by suggesting that the promotion of IK is fronted by those intending to appeal to the hearts and minds of ordinary people and their sympathisers alone, instead of even the critics of IK, and arguing that it is empty and valueless fans the flames of continued peripherisation of this key resource. However, it is also plausible to analyse the concept of IK critically, and to pinpoint why it is identified as rhetoric.

In addition to referring to ‘the *rhetoric* and practice of indigenous knowledge’, Agrawal (1995:416) questions what it is that really ‘distinguishes indigenous from western knowledge’. Whilst of course these questions are pertinent to understanding the value of IK, it can be argued that this tendency to look for the dominant type of knowledge – be it indigenous or scientific – contributes to the marginalisation of the other, and does little to promote the hybridisation of these two types of knowledge.

### Marginalisation of indigenous knowledge

IK has been on the periphery for centuries, especially as development and DRM practitioners prefer scientific knowledge. This act of ‘privileging one form of knowledge over another’ (Nygren 1999:271) has made it difficult for proponents of IK to promote its utilisation in development planning, and ‘has retarded its development and integration’ (Ocholla 2007:3). In fact, IK is seen mainly as inefficient, primitive, archaic, inferior and inefficient (Kelkar 2007), an obstacle to development (Agrawal 1995) or a constraint on progress (Nygren 1999). Nyong *et al.* (2007:788) lament the fact that ‘little has been done to incorporate [*IK*] into formal climate change mitigation and adaptation strategies’ despite its demonstrated importance in climate-change resilience debates.

Viewing it as an obstacle to development has resulted in IK being ‘often ignored, disregarded or deemed insufficient for [*disaster risk management*]’ (Mercer 2012:99). This is mainly because DRM practitioners view Western knowledge as having an edge over IK as it is universal and can be employed to theorise solutions to similar problems, whilst IK is viewed as ‘closed, parochial, unintellectual, primitive and emotional’ (Briggs 2005:102) and location-specific (Briggs 2005; Mercer 2012).

In the Zimbabwean context, Shizha (2006:22) concurs with the findings of the research in Buhera and Chikomba where colonialism was identified as a factor contributing to the marginalisation of IK, arguing that during the colonial era, IK was often ‘discounted as invalid and irrelevant in contemporary Africa’ and viewed as ‘undesirable and subordinate’. Shizha goes on to argue that the two types of knowledge are not accorded the same level of respect, especially by policy makers and the education system, probably because of a Western knowledge-biased teacher training system in the country.

Briggs (2005) and Ngulube *et al.* (2011) view the marginalisation of IK as highly unjustified. They argue that critics tend to focus more on the misgivings related to IK, yet there is not much criticism of Western knowledge – which, as argued by Briggs (2005), has failed to inform effective development and livelihood transformation in Africa. Ngulube *et al.* (2011) even argue that the lack of documentation of IK by the national knowledge repository in Zimbabwe, the National Archives of Zimbabwe, is a form of injustice perpetrated on the holders of such knowledge.

Knowledge marginalisation is gendered too, as knowledge generated and communicated especially by women is usually underrated, despite women possessing ‘particularly rich insights in many indigenous cultures and local knowledge systems’ (Agrawal 1995:417). Kelkar (2007:301) opines that there exist ‘different knowledges constructed by men and women as a result of their differential roles, access to and rights over resources, and work patterns’. It is further argued that rural women interact with the natural environment more than their male counterparts, since they draw their livelihoods from that natural environment, whilst most economically active men are engaged in paid work in urban areas. It is unfortunate that the undermining of women is prevalent, especially in patriarchal societies such as those existing in rural Zimbabwe, and this extends even to the knowledge that they possess and how that knowledge is communicated. The question of whose knowledge dominates is further discussed in subsequent sections.

### Indigenous knowledge: The challenges

Proponents of IK have identified modernisation, Christianity and colonialism as some of the reasons why belief in IK is low (Mawere 2010; Ngulube *et al.* 2011; Shizha 2006). As the research discovered, there is an increasing dislike of some cultural practices that were previously used to generate IK, such as *mikwerera* (rainmaking ceremonies), whilst in some cases these traditional practices have been discontinued. This is influenced particularly by changing beliefs and modernisation, which has also led to the further marginalisation of the little IK in existence in the researched districts.

Just as culture is tied to knowledge development, language also cannot be divorced from the creation and communication of knowledge, as it ‘provides the context within which we are able to know’ and ‘while constructing knowledge, we are processing cognitions through language’ (Renzl 2007:45). The low uptake of vernacular languages, especially in Zimbabwe's education system, could spell doom for future knowledge development. For example, when Dambudzo Marechera, a 1980s revered Zimbabwean writer from the Shona tribe, was responding to whether he had ever thought of writing in his Shona vernacular, he responded (Marechera 2009):

It never occurred to me. Shona was part of the ghetto daemon I was trying to escape. Shona had been placed within the context of a degraded, mind-wrenching experience from which apparently the only escape was into the English language and education. (p. 6)

This can be seen as symptomatic of the general dislike and disregard of anything involving vernacular languages in Zimbabwe, possibly because of the superiority accorded to the English language by the then colonial administration, a situation that has been perpetuated even by the postcolonial governments. Such continued disregard of vernacular languages might lead to the extinction of indigenous communities’ knowledge.

Socio-economic change has also contributed to dwindling belief in and usage of IK. Fleuret (1986) puts the argument more succinctly, arguing that:

Knowledge of wild and famine foods is disappearing; food preservation techniques are abandoned … agricultural strategies are affected by land reform and introduced varieties, while institutions of food and labor sharing are curtailed by the development of cash markets in these commodities. (p. 227)

In the case of Zimbabwe, the production of traditional crops or famine foods has significantly diminished, particularly because of the creation of a ‘maize culture’, as maize is one of the main cash crops in the country. The production of small grains has also been hampered by the absence of technology to reduce the labour burden of food processing on resource-constrained households as well as dwindling knowledge of traditional food processing.

One of the main challenges that IK faces is its continued lack of documentation; documentation would have contributed to its testing and validation over a period of time. Ngulube *et al.* (2011) attribute the dearth of IK to its lack of documentation at national level, whilst attrition of the older population contributes to its potential extinction, as the undocumented knowledge dies with the beholder. The formulation of a clear policy that makes documentation of IK a systematic nationwide project is a necessity, as without such a national policy an uncoordinated approach to documentation might result, possibly leading to misinterpretation and further marginalisation of the knowledge.

In addition, it is important that even though the documentation of IK might be viewed from the realm of a national project, this is not to assume that there is a ‘national IK’ as such, as implied by Ngulube *et al.* (2011:217) when they argue for the ‘importance of recording national IK’ as IK is largely known to be place-specific (Briggs 2005; Mercer 2012). This means that even within a country, differential IK may exist. However, communities in Buhera and Chikomba appeared to possess more or less the same IK, probably due to the districts’ geographical proximity.

As arguments for IK documentation continue to mount, this presents challenges regarding the selection of the IK to preserve. Ngulube *et al.* (2011:274) ask ‘what [*IK*] do we preserve and what [*IK*] do we allow to disappear?’ Another challenge relates to the capacity ‘to identify, collect, and develop indigenous knowledge into contemporary, useable formats’ (Williams & Muchena 1991:55). Possibly one important approach would be for the educational curriculum, regardless of discipline, to integrate IK (Ma Rhea 2004), because IK is a multidisciplinary resource not just restricted to the social sciences. In addition, access to IK remains a challenge. It is argued that ‘indigenous knowledge is not always equally shared or accessible to all local residents’ (Hart & Mouton 2005:254) and that access depends on the power dynamics prevalent in society, where it is usually the ‘second-class citizens’ of society that are marginalised.

The location-specific nature of IK, which makes it difficult for the knowledge to be integrated in national policy making, has also been seen as one of the challenges that its proponents face in promoting its widespread usage (Briggs 2005; Mercer 2012). The prevailing argument has been that development and DRM practitioners cannot rely on IK to theorise and develop interventions that can be replicated in other areas. In addition, it cannot be assumed that ‘the knowledge generated by one farmer is the knowledge of all the other farmers’ (Hart & Mouton 2005:255), even though the farmers might be from the same locality.

### Cultural dominance and knowledge production

It is very difficult to discuss IK without discussing issues of culture, as there exists a strong umbilical cord tying knowledge production to a people's culture (Shizha 2006). Williams and Muchena (1991:55) argue that ‘vital information on natural resource management ensuring sustainability is often encoded in unique forms such as proverbs, myths, rituals, and ceremonies’. For example, ceremonies such as *mikwerera* are key to producing IK that has sustained livelihoods in communities such as Buhera and Chikomba.

The importance of a type of knowledge is linked to the importance of a particular culture or society that developed that knowledge. As such, if a certain culture is ‘seen more as an impediment to understanding and effective action’ (Hewitt 2012:85), the knowledge that such a culture produces will be disdained. It is, therefore, essential to understand cultural dynamics, as this helps in establishing what makes one knowledge type dominant over the other (Hewitt 2012).

Zimbabwean society is not homogenous, as is the case in any other country. As such, IK produced in the country should be valued regardless of the cultural group that produced it. The broad Shona culture, if there is ever a homogenous one (considering the wide range of ethnic groups within the Shona tribe) should never be seen as superior to the Ndebele culture (again, if there is a homogenous one), and vice versa, and neither of these should be viewed as inferior to foreign cultures. For example, rain ceremonies are not unique to the Shona culture alone, but are also practised by a variety of indigenous groups around the world. In fact, research has shown that ‘people often appeal to deities when coping with severe stress’ (Chester *et al.* 2012:109). All the relevant stakeholders should play a key role in preserving all Zimbabwean cultures, as well as putting measures that will diffuse cultural dominance and accord IK some level of integrity. As long as we have a problem of according superiority to certain cultures, viewing others as backward and primitive (Nygren 1999), the challenge of IK marginalisation will continue to dog us.

Whilst cultural dominance determines whose knowledge is superior (Hewitt 2012), Mercer (2012:98) argues that ‘political, economic and social power in society determine “whose knowledge counts”’. Power dynamics within one culture can also hinder effective production and communication of knowledge as some of these processes are sometimes ‘controlled and directed by small, powerful elites’ (Hewitt 2012:94). Relating this to research design, the principle that researchers should consult traditional leaders sometimes leads to knowledge from those leaders being viewed as more valuable and accurate than that produced and retained by the ‘subalterns in society’. Even when researchers employ sampling techniques such as snowballing, they will still be directed to the powerful elites in society, whilst the economically, politically and socially downtrodden will remain on the periphery of knowledge creation processes. The same applies to knowledge produced and held by women in patriarchal societies (Agrawal 1995; Kelkar 2007; Ramphele 2004).

Nygren (1999:268) cements arguments on the social construction of knowledge, stating that ‘all knowledges are socially constructed [*and*] the focus of analysis should be on those processes that legitimize certain hierarchies of knowledge and power between local and global (scientific) knowledges’. The marginalisation of IK in favour of scientific knowledge can therefore be linked to its social construction, whilst the power dynamics within any grouping also determine whose knowledge will be the point of reference.

Despite the existing marginalisation and near extinction of IK, and the challenges that it faces, some IK is still in use and has significantly helped in sustaining rural agro-based livelihoods, especially in the Buhera and Chikomba districts in Zimbabwe. As such, it is essential to marry this knowledge with modern science in a process that will see each type offsetting the weaknesses of the other (Mutasa 2013).

### Indigenous knowledge: Policy options

Although the IK discourse has been in existence for decades, dating back to the 1950s when IK was savagely discredited as an obstacle to development (Agrawal 1995), there has been an increase in voices supporting its incorporation into development planning since scientific knowledge has not been failure-proof either (Briggs 2005). Kelkar (2007) refers to the shift in development thinking influenced by a failure mainly of grand development theories and of the ‘transfer of technology model’ as reasons why IK has attracted such a focus from development practitioners. Ngulube *et al.* (2011) opine that it is high time the documentation of IK is taken seriously, before its complete extinction. For this to be achieved, they propose the establishment of IK centres (IKCs) that will act as ‘repositories of community knowledge, places where knowledge can grow, and places where two-way cultural learning can occur’ (Ngulube *et al.* 2011:270).

Whilst the need for community and national repositories of IK is plausible, establishment of such centres will be futile in the absence of a legal framework that constitutes such institutions. The *National Library and Documentation Service Act* (Act no. 11 of 1985), for example, was integral to the establishment of the National Library and Documentation Service, whilst the *National Archives of Zimbabwe Act* (Act no. 8 of 1986) facilitated the setting up of the National Archives. As such, the establishment of IKCs needs a supporting legal framework, probably in the mould of a *National Indigenous Knowledge Documentation and Preservation Act*, advocating for the establishment of such centres and engaging in a nationwide IK documentation exercise. There should also be provisions in the legal framework setting out how such initiatives will be supported financially, particularly from the National Treasury. It might be prudent too if the IKCs are to work in unison with the National Archives, parliamentary constituency information centres, information kiosks, libraries and cultural centres already in existence in most areas around Zimbabwe.

In addition to the establishment of such centres, the development of IK documentation and preservation skills is also a necessity if a nationwide project on documenting and preserving IK in Zimbabwe is to be successful (Williams & Muchena 1991). This is very key and urgent, considering that the remaining elderly, who are the custodians of the majority of IK, are struggling to pass the knowledge on to an already non-receptive young generation that is heavily blinded by their dislike for the knowledge and traditions credited for IK production.

Arguments for the integration of IK into the agricultural education curriculum have been growing for several decades, especially as IK has been utilised for years to sustain dryland agricultural production. Williams and Muchena (1991:55) argue that educators should start ‘with the farmers’ indigenous knowledge, [*and*] move from the familiar to the unfamiliar, from the concrete to the abstract in the process of promoting sustainable agriculture’. This should involve embracing IK particularly in curriculum development, revision and delivery, and not propping up one knowledge type over the other, as has been the case with Western-based education, that has often ‘been criticised for dismissing and attempting to supplant indigenous knowledge’ (Ma Rhea 2004:7).

### An argument for knowledge hybridisation

The importance of IK as a resource for formulating survival strategies and development planning cannot be underestimated. According to Carr and Kettle (2009:132), IK ‘cannot be ignored or diminished in any study of the emergence of drought-related crises’. As such, it is important that IK is incorporated into scientific rainfall predictions (Mutasa 2011). The fact that communities in Buhera and Chikomba have utilised their IK to predict rainfall patterns and inform agricultural planning in the absence of scientific rainfall predictions, coupled with their poor access to and interpretation of weather data from the Meteorological Services Department, is evidence enough of the importance of IK in smallholder agriculture, and the trust that the communities have invested in such knowledge. As such, arguments for marrying the two knowledge types so that farmers can ‘benefit from both worlds’ cannot be dismissed as far-fetched.

In fact, arguments for knowledge hybridisation are not new to the IK discourse as, according to Bohensky and Maru (2011), such arguments were high on the agenda in the 1990s. They further argue that despite the challenges that the ‘project of integration’ faces, there is a need to ensure that the different types of knowledge maintain their identities but continue to be strengthened through interaction with other knowledge types. Shizha (2006:28) argues that knowledge hybridisation aims to achieve a state whereby ‘indigenous and Western knowledges condition one another’. Such hybridisation does not aim to treat either knowledge as superior but ‘builds upon the strength of both inside and outside knowledge’ (Mercer 2012:99). Mercer (2012) further argues that:

[*Rather*] than allowing one form of knowledge to dominate, as in the case of outside knowledge, or weighing up one kind of knowledge against the other, … there should be an effort to ‘bridge the gap’ or to ‘reconcile science and tradition’. (pp. 101–102)

This offsetting of weaknesses brought through the hybridisation of knowledge has been influential in sustaining livelihoods in times of adversity, especially in smallholder farming communities in rural Zimbabwe. However, hybridisation is often seen as not planned, since most people in the farming communities do not make clear distinctions between inside and outside knowledge, probably because inside knowledge, due to its dynamic nature and fluidity (Briggs 2005; Mercer 2012), has incorporated some attributes of outside knowledge, making it a useful resource in constantly changing societies and environments. In fact, as Mercer (2012:99) argues, knowledge hybridisation results in inside knowledge being ‘shaped by outside knowledge in its continuous evolution’. Additionally, Briggs (2005:104) opines that ‘farmers do not think of knowledge as coming from two or more separate, self-contained and competing systems anyway’.

Knowledge hybridisation is not bereft of challenges though. Mercer (2012:104–105) and Briggs (2005:103) argue that the following are potential barriers to the smooth integration of outside and local knowledge:

- The perceived superiority of outside knowledge over local knowledge.
- The context-specific and embedded nature of local knowledge, whilst Western knowledge aims to be universal.
- The laborious process of co-production and utilisation of hybrid knowledge aiming at making it accessible, understandable to and usable by everyone.

It is, therefore, important that these challenges are acknowledged and solutions are sought. For example, marrying the two types of knowledge should be done *in situ* ‘as divorce from context makes the [*indigenous*] knowledge irrelevant and invalid’ (Kelkar 2007:298).

## Conclusion

The continued promotion of ‘new traditions’ introduced by postcolonial modernisation and Christianity at the expense of traditional practices such as holding rainmaking ceremonies can be viewed as the lubricant greasing the machinery marginalising IK in Zimbabwe. It is unfortunate that regardless of the value that Zimbabwe's rural smallholder farmers accord to their IK, scientific knowledge will continue to be the preferred choice unless there is a systematic approach to documenting, validating, preserving and communicating IK, as it is a useful tool in dryland smallholder agriculture.

This article argued that there are some similarities between indigenous and scientific rainfall predictions. As such, it is important that the proponents of knowledge hybridisation identify these similarities, ensure documentation of the similarities and differences, and identify ways of facilitating the incorporation of different knowledge types into agricultural planning and DRM.

A legal framework in the mould of a *National Indigenous Knowledge Documentation and Preservation Act* might also be necessary to facilitate IK documentation through the establishment of relevant institutions and mechanisms. In the absence of such a legal framework, it will be difficult to counteract the peripherisation of IK, whereas IK has been very influential, especially in the context of DRM in rural Zimbabwe.

### Recommendations

IK is a key resource in agricultural planning and DRM, as discovered from the research in Buhera and Chikomba districts of Zimbabwe. It is important, therefore, that:

- IK be documented, validated, preserved and communicated so that it can inform agricultural processes in farming communities. This involves developing and implementing the necessary legal framework supporting the creation of structures and mechanisms to support the processes.
- The use of IK be promoted nationally to counteract its possible extinction.
- IK be integrated into the education curriculum, particularly in agricultural training and research.
- IK and scientific knowledge be synchronised in a way that they can offset the weaknesses of the other in sustaining rain-fed agriculture and livelihoods of smallholder farmers in rural Zimbabwe.
